# Rewiring neuronal microcircuits of the brain via spine head protrusions-a role for synaptopodin and intracellular calcium stores

**DOI:** 10.1186/s40478-016-0311-x

**Published:** 2016-04-22

**Authors:** David Verbich, Denise Becker, Andreas Vlachos, Peter Mundel, Thomas Deller, R. Anne McKinney

**Affiliations:** Integrated Program in Neuroscience, McGill University, Montreal, QC H3G 0B1 Canada; Institute of Clinical Neuroanatomy, Neuroscience Center, Goethe-University Frankfurt, D-60590 Frankfurt/Main, Germany; Department of Pharmacology and Therapeutics, McGill University, Bellini Life Sciences Building, Room 167, Montreal, QC H3G 0B1 Canada; Harvard Medical School and Department of Medicine, Massachusetts General Hospital, Boston, MA 02114 USA; Present address: Department of Fundamental Neurosciences, University of Lausanne, 1005 Lausanne, Switzerland; Present address: Institute of Anatomy II, Faculty of Medicine, Heinrich-Heine-University, D-40225 Düsseldorf, Germany

**Keywords:** Dendritic spines, Synaptopodin, Calcium, Structural plasticity, Ryanodine

## Abstract

Neurological diseases associated with neuronal death are also accompanied by axonal denervation of connected brain regions. In these areas, denervation leads to a decrease in afferent drive, which may in turn trigger active central nervous system (CNS) circuitry rearrangement. This rewiring process is important therapeutically, since it can partially recover functions and can be further enhanced using modern rehabilitation strategies. Nevertheless, the cellular mechanisms of brain rewiring are not fully understood. We recently reported a mechanism by which neurons remodel their local connectivity under conditions of network-perturbance: hippocampal pyramidal cells can extend spine head protrusions (SHPs), which reach out toward neighboring terminals and form new synapses. Since this form of activity-dependent rewiring is observed only on some spines, we investigated the required conditions. We speculated, that the actin-associated protein synaptopodin, which is involved in several synaptic plasticity mechanisms, could play a role in the formation and/or stabilization of SHPs. Using hippocampal slice cultures, we found that ~70 % of spines with protrusions in CA1 pyramidal neurons contained synaptopodin. Analysis of synaptopodin-deficient neurons revealed that synaptopodin is required for the stability but not the formation of SHPs. The effects of synaptopodin could be linked to its role in Ca^2+^ homeostasis, since spines with protrusions often contained ryanodine receptors and synaptopodin. Furthermore, disrupting Ca^2+^ signaling shortened protrusion lifetime. By transgenically reintroducing synaptopodin on a synaptopodin-deficient background, SHP stability could be rescued. Overall, we show that synaptopodin increases the stability of SHPs, and could potentially modulate the rewiring of microcircuitries by making synaptic reorganization more efficient.

**Electronic supplementary material:**

The online version of this article (doi:10.1186/s40478-016-0311-x) contains supplementary material, which is available to authorized users.

## Introduction

Functional and structural deficits associated with neurological diseases can be consequences of disruptions in the neuronal microcircuitry [1, 2]. Following axonal denervation, the affected network responds to the changes in synaptic transmission and perhaps other resulting injuries with profound reorganization [2–7]. In connected and denervated regions reactive synaptogenesis and collateral sprouting occurs, which can, in part, reestablish the balance of excitation and inhibition and the flow of information [2, 5, 7, 8]. In non-denervated regions, changes in network activity may result in the rewiring of the existing circuitry, which allows the brain to reroute information through neighboring areas, thereby bypassing the damaged region [8–10]. This post-lesional rewiring of intact circuits is important therapeutically, since it can partially recover functions [11, 12] and can be further enhanced using novel therapeutic [7, 8, 13–15] or modern rehabilitation strategies [16–18].

The cellular mechanisms for structural microcircuit reorganization are not yet fully understood, but local synaptic rewiring is a clear outcome. In particular dendritic spines appear to be capable of rewiring neurons, since they exhibit a considerable structural plasticity in response to external cues, such as glutamate, that allows them to remodel their geometry and connectivity [19–23]. We have investigated the role of spines in the rewiring of microcircuits in previous studies and have reported that a subset of innervated spines is able to form spine head protrusions (SHPs), which can form new synapses with neighboring but not yet connected boutons [24, 25]. This phenomenon became much more frequent after exposure of the neurons to the action potential blocker tetrodotoxin (TTX), demonstrating that perturbations in network activity can enhance this mechanism of microcircuit reorganization. Indeed, glutamate release from neighboring axon terminals appears to regulate SHP formation and stabilization [24]. Once formed, SHPs grow towards active glutamate sources, suggesting that they are part of a mechanism that allows modifications of an established microcircuit in an activity-dependent manner [24, 25].

Since the cellular and molecular mechanisms involved in SHP formation and stabilization are possible targets to enhance and support the rewiring of networks, we searched for candidate regulatory molecules. Synaptopodin appeared to be a promising candidate regulatory molecule in this regard, since it is an actin-associated protein [26] found in a subpopulation of mature spines [27, 28], and is involved in both functional and structural synaptic plasticity [29–31]. Moreover, it is an essential component of the spine apparatus organelle [28, 32, 33], a local Ca^2+^ store of spines [29, 34, 35]. Using live-imaging and transgenic approaches, we investigated whether synaptopodin is involved in SHP formation and stabilization. Although synaptopodin-loss does not affect the formation of SHPs, synaptopodin-loss leads to SHPs that are transient and unstable. Together with pharmacological experiments, we suggest that Ca^2+^ release from synaptopodin-associated stores is required for the stability of SHPs.

## Materials and methods

### Slice cultures

Organotypic hippocampal slice cultures were prepared at P3-8 from mice (see below for strains) of both sexes using either the roller-tube method [36] or the interface method [37], as described previously. See Additional file 1: *SI Materials and Methods* for further details. All experimental manipulations were carried out after 3 weeks in vitro to ensure the reestablishment and stabilization of synaptic structures and functions in the organotypic slice cultures.

### Mouse strains

L15 [38] GFP expressing mice were used as wild type, SP-KO [32] and SP-KO mice expressing GFP-tagged synaptopodin [39] were previously described. See Additional file 1: *SI Materials and Methods* for further details.

### Immunostaining and static imaging

Slice cultures were immunostained as described previously [40].

### Time-lapse confocal imaging

Live confocal imaging was carried out essentially as previously described [25]. See Additional file 1: *SI Materials and Methods* for further details.

### 3D Image reconstruction and analysis

Image stacks were deconvolved using Huygens Essential software (Scientific Volume Imaging, Hilversum, The Netherlands) with a full maximum likelihood extrapolation algorithm. Volume rendering and quantification were carried out using Imaris ×64 software (Bitplane AG, Zurich, Switzerland). No filtering or resampling was performed. SHPs were quantified by finding pointy structures emerging from spine heads that were ≥0.5 μm in length. To calculate SHP lifetime, every SHP that appeared *de novo* on a spine was counted and if SHPs appeared in only one time frame, then it was assumed that these SHPs had lifetimes corresponding to the interval between *z* stacks (2 or 5 min). Because stack acquisition took ~30 s (usually 24 stacks for 60 min imaging experiment, with a time interval of 2 min), the theoretical maximum lifetime is 48 min (48 min of imaging, not counting the 12 min of image acquisition). See Additional file 1: *SI Materials and Methods* for further details.

### Electrophysiology

Whole-cell voltage clamp recordings were obtained from CA1 pyramidal neurons in either wild type or SP-KO slices held at − 60 mV with an Axopatch 200A amplifier (Molecular Devices), as previously described [41]. See Additional file 1: *SI Materials and Methods* for further details.

### Statistics

All values are given as the mean ± SEM. Normality of data distribution was determined with Kolmogrov-Smirnov test. Statistical comparisons were made with two-tailed, two sample or paired *t* tests or one-way ANOVA with Dunnett’s test to compare multiple treatments to a treatment of interest where appropriate, and Mann-Whitney tests were used for non-parametric testing where appropriate and *P* < 0.05 considered as significant for all statistical comparisons.

## Results

### Spines that form SHPs regularly contain synaptopodin

**Fig. 1:**
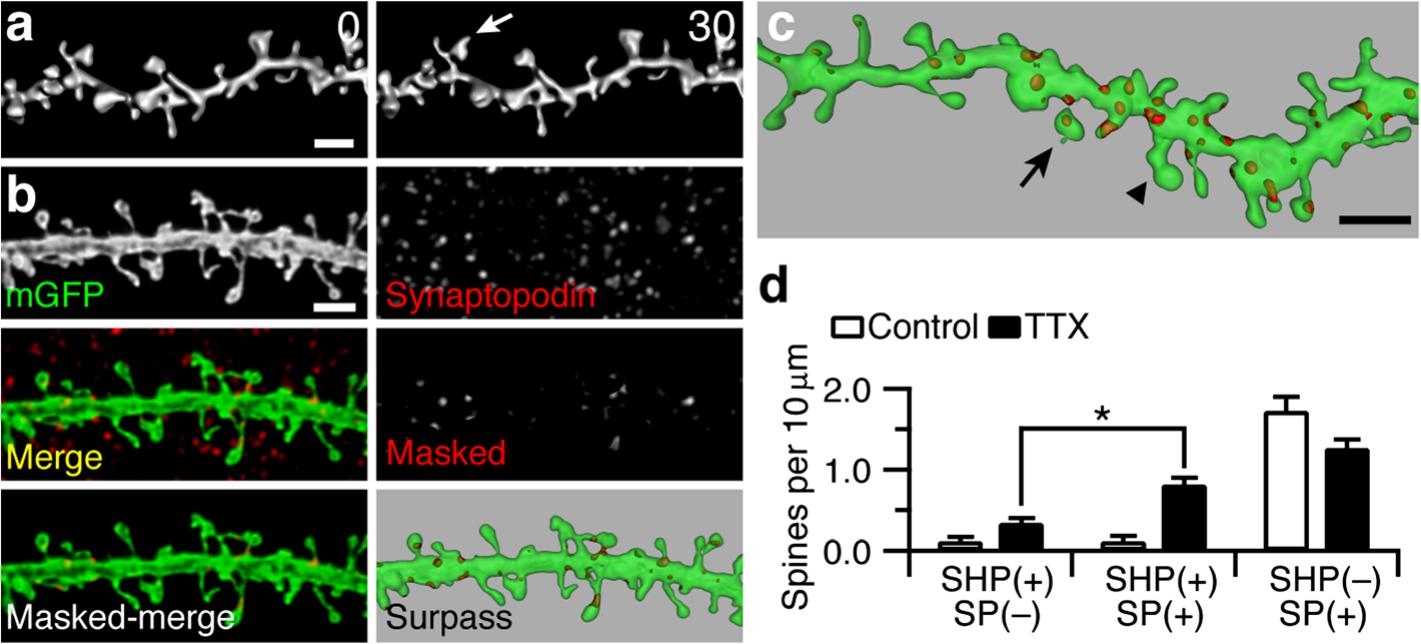
Spines that form SHPs regularly contain synaptopodin. **a** Exposure of hippocampal slice cultures to 1 μM TTX leads to the formation of SHPs within 30 min. Typical tertiary dendrite from a cultured CA1 pyramidal neuron rendered in 3D. Arrow points to a SHP. Time is in minutes in top-right corner. **b** Immunostaining and image analysis for synaptopodin in slice cultures. Synaptopodin (*red*) was found in dendritic spines as well as dendritic shafts of CA1 neurons expressing membrane GFP (mGFP; *green*). Image is a maximum intensity projection of 8 consecutive *z* sections. The green channel was then used to mask the red channel to isolate only synaptopodin-positive puncta within the dendrite of interest (*Masked*; *Masked-merge*). The remaining red puncta and mGFP were reconstructed in 3D (*Surpass*, Imaris). **c** Example of a dendrite (*green, semi-transparent*) with synaptopodin-positive puncta (*red*) in spines with SHPs (*arrows*) and a spine with a SHP lacking synaptopodin (*arrowhead*). Scale bars in all panels, 2 μm. **d** Quantification of spines with SHPs (SHP (+)) either positive or negative for synaptopodin (SP) after 2 h treatment of either control (SP (+), 0.12 ± 0.07; SP (–), 0.12 ± 0.05 spines per 10 μm of dendrite; *n* = 12 branches from 6 slices, 338 μm of dendrite; not significant, paired *t* test) or TTX-containing medium (SP (+), 0.81 ± 0.09; SP (–), 0.34 ± 0.06 spines per 10 μm of dendrite; *P < 0.001 paired *t* test; *n* = 30 branches from 8 slices, 791 μm of dendrite) and of spines with synaptopodin (SP (+)) without SHPs (SHP (–)) after 2 h treatment of either control or TTX (control, 1.72 ± 0.18; TTX treated, 1.27 ± 0.10 spines per 10 μm of dendrite)

Synaptopodin serves as a useful and consistent surrogate marker for the dendritic spine apparatus [27, 28], a local Ca^2+^ store [29, 34, 42], and loss of synaptopodin abolishes the formation of spine apparatuses [32]. As 20–30 % of CA1 spines contain a spine apparatus [33] and synaptopodin [27, 28], we reasoned that spines may need the spine apparatus/synaptopodin to form SHPs. To determine whether dendritic spines that formed SHPs bore synaptopodin, we first immunostained mature slice cultures (≥3 weeks in vitro) of mGFP mouse hippocampus for synaptopodin. We either treated slices with control culture media or culture media with 1 μM TTX for 2 h (to increase the number of SHPs) and then fixed and immunostained cultures for synaptopodin. Similar to previous reports [27, 35, 39], in vitro synaptopodin staining was punctate (Fig. 1a, b, c) and located in CA1 dendrites and some spines. We found that control slices, i.e., slices that were not treated with TTX, had similar numbers of SHP-containing spines that were synaptopodin-positive or synaptopodin-negative (Fig. 1d). After 2 h of TTX treatment, however, the total number of SHPs increased and now significantly more SHP - containing spines with synaptopodin puncta were found (Fig. 1c, d). These results provide initial evidence for a link between synaptopodin and SHPs. However, not all synaptopodin-positive spines formed a SHP (Fig. 1d), suggesting that the presence of synaptopodin in a spine is not sufficient to predict the formation of a SHP.

### SP-KO slice cultures have similar morphological and physiological properties as wild type slice cultures

To provide further evidence for a link between synaptopodin and SHPs, we analyzed the morphological and physiological properties of CA1 neurons in synaptopodin-deficient (SP-KO) mice. Analysis of these mice previously revealed that synaptopodin is not involved in the regulation of spine number [32], but rather in the regulation of spine head growth under conditions of plasticity [30]. Since these data point to an involvement of synaptopodin in structural spine remodeling and synaptic plasticity, we wondered whether spines of CA1 neurons of SP-KO might differ in their ability to form SHPs from spines of wild type animals. To obtain a baseline for these experiments, we crossbred SP-KO mice with a mouse line expressing mGFP in neurons [38] and studied the morphological and functional properties of mGFP-positive CA1 neurons in wild type and SP-deficient cultures.

Consistent with a previous in vivo report [32], mGFP-positive CA1 pyramidal cells in organotypic slice cultures had a similar morphology in both wild type and SP-KO slices and *synaptopodin* deletion did not affect mean spine density (wild type, 1.71 ± 0.08 spines per μm of dendrite; SP-KO, 1.74 ± 0.06 spines per μm of dendrite), mean spine length (wild type, 1.35 ± 0.03 μm; SP-KO, 1.39 ± 0.03 μm) or mean spine volume (wild type, 0.54 ± 0.01 μm^3^; SP-KO, 0.54 ± 0.01 μm^3^) (Additional file 2: Figure S1a-c). We then recorded mEPSCs to ensure that if we observed any differences in SHPs between SP-KO and their controls (Additional file 2: Figure S1d), these could not be explained by changes in excitatory neurotransmission. We found that the average amplitude (Additional file 2: Figure S1e, f *left*), inter-mEPSC interval (Additional file 2: Figure S1e, f *right*) and decay time (wild type, 2.57 ± 0.08 ms; SP-KO, 2.77 ± 0.16 ms) of mEPSCs were comparable in wild type and SP-KO slices. Further, in SP-KO hippocampus, afferent inputs to CA1 are comparable between SP-KO and wild type mice [30, 32]. Thus, we find that culturing slices from SP-KO mice crossed with mice expressing mGFP does not change basal morphological properties or excitatory neurotransmission onto CA1 pyramidal cells.

### SHPs have shortened lifetimes in SP-KO hippocampal slices

**Fig. 2:**
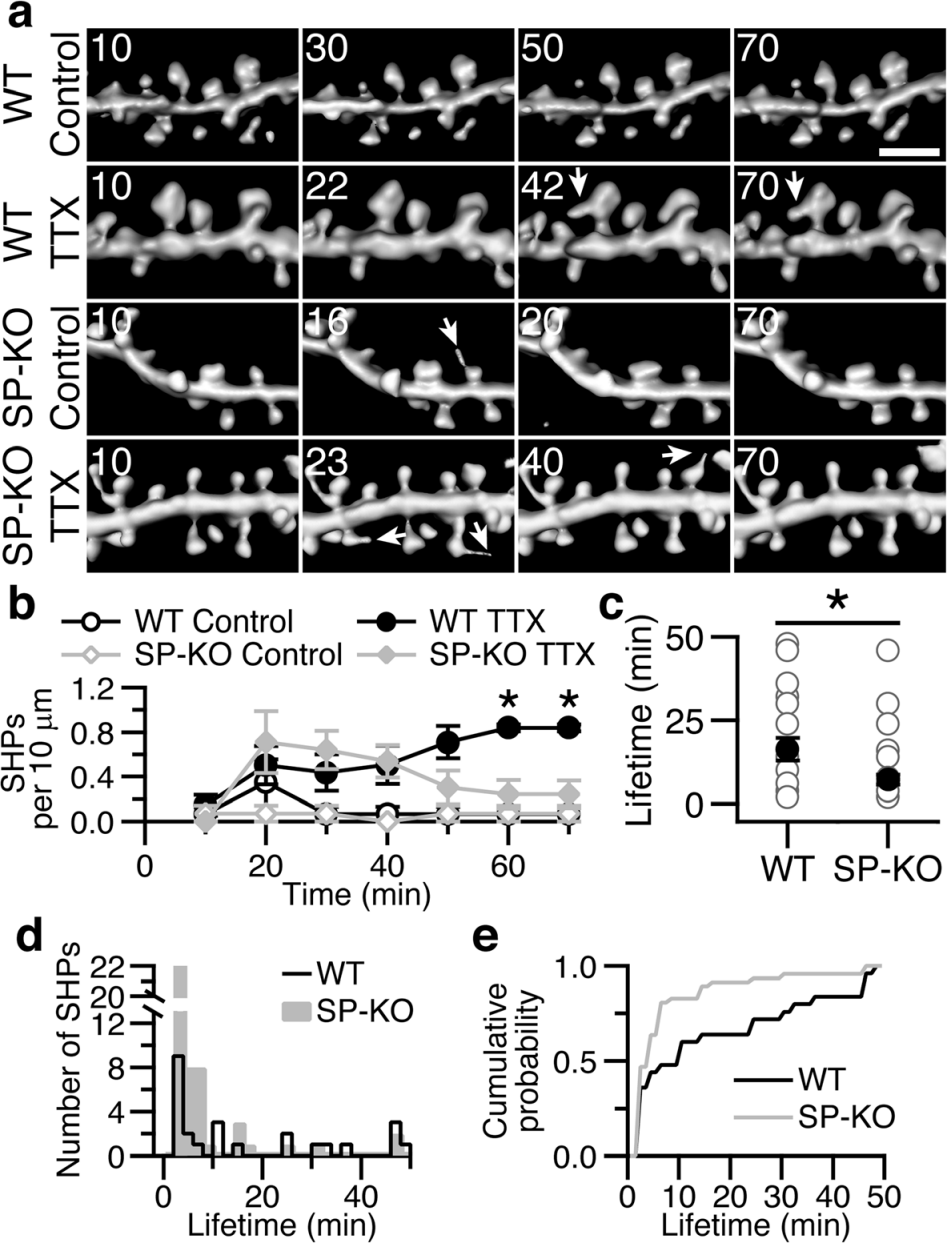
SHPs have shortened lifetimes in SP-KO hippocampal slice cultures. **a** Examples of small regions of dendrites from WT (*top two rows*) and SP-KO (*bottom two rows*) exposed to 60 min of either control or TTX-containing medium (as indicated). Arrows denote SHPs. Time is in minutes in top-left corner, and 10 min was the starting time point. Scale bar, 2 μm. **b** Time course of SHP formation in WT (*black symbols*) or SP-KO (gray symbols) slices exposed to control solution (*open symbols*) or TTX (*closed symbols*). Although SP-KO dendritic spines can form SHPs, they are unstable and retract, while WT spines can form protrusions that persist. *n* = 6 slices, ~150 μm of dendrite per treatment. *P < 0.05, two-tailed, one-way ANOVA with Dunnett’s test compared with WT control, SP-KO control and SP-KO TTX treated. **c** Quantification of mean lifetime of all SHPs (from both of control or TTX experiments) from WT and SP-KO dendrites. SHPs had a mean lifetime of 16.32 ± 3.40 min in WT (*n* = 25 SHPs from 12 slices), compared to 7.15 ± 1.48 min from SP-KO (*n* = 47 SHPs from 12 slices). Gray circles are individual lifetimes of SHPs, and black circles are mean lifetimes ± SEMs. *P < 0.05, Mann-Whitney. **d** SHPs sorted by lifetime in WT (*black framed cityscape*) and SP-KO (*gray bars*). **e** Cumulative distribution of all SHP lifetimes from WT (*black line*) and SP-KO (*gray line*) slices

We next turned to live imaging experiments to study the formation and stability of SHPs in cultures of SP-KO and wild type mice. In wild type slice cultures, 0.07 ± 0.07 SHPs per 10 μm of dendrite were present after an hour in control medium (Fig. 2a, b). Treating cultures with TTX for an hour increased the number SHPs to 0.84 ± 0.03 SHPs per 10 μm of dendrite (Fig. 2a, b). The average lifetime of the SHPs formed in wild type slices was 16.32 ± 3.40 min (Fig. 2c, from both control and TTX treated; lifetimes not significantly different between control and TTX-treated groups, *P* > 0.05, Mann-Whitney test). Thus, the preponderance of SHPs in wild type slices were maintained for ≥15 min. Figure 2b shows the time course of SHP formation over the imaging period and shows that in wild type slices, SHPs accumulate as the imaging session proceeds.

Based on our immunostaining finding that only few spines without synaptopodin formed SHPs, we reasoned that SP-KO slices would form few SHPs. Surprisingly, we found that SHPs formed in SP-KO slices as they did in controls, but these SHPs were unstable and their lifetimes were considerably shorter (Fig. 2a-c). On average, SHPs formed in SP-KO slices had a mean lifetime of 7.15 ± 1.48 min (from both control and TTX-treated slices) that was significantly shorter than mean wild type lifetime of 16.32 ± 3.40 min (Fig. 2c, d). In SP-KO slices exposed to control medium, 0.07 ± 0.07 SHPs per 10 μm of dendrite were found after 1 h (Fig. 2a, b). The addition of TTX to the medium increased SHP formation initially, but most SHPs retracted within 10–15 min of their formation so that the number of SHPs after 1 h was not significantly different from SP-KO and wild type slices treated with control solution (0.24 ± 0.13 SHPs per 10 μm of dendrite; Fig. 2a, b). Moreover, analyzing the distribution of SHP lifetimes clearly shows that SP-KO SHPs have shortened lifetimes (Fig. 2d, e). Thus, SHP kinetics on SP-KO neurons are different from the majority of SHPs found in wild type slices. In summary, although SHPs can form on SP-KO spines, these SHPs are unstable and retract quickly.

### Ca^2+^ release from ryanodine-sensitive stores increases SHP lifetime

After demonstrating the link between synaptopodin and the stability of SHPs, we wondered how synaptopodin could exert its effect. In previous work, we have shown that synaptopodin is an essential component of the spine apparatus [32], a specialized form of smooth endoplasmic reticulum (ER) that is in contact with both the dendritic ER as well as the postsynaptic density [43]. The spine apparatus has been suggested to function as a local Ca^2+^ store that could regulate actin-based spine motility by releasing or sequestering Ca^2+^ from/into the smooth ER [29, 34, 35, 44]. This Ca^2+^ release likely occurs through ryanodine-sensitive receptors found throughout dendritic and spine ER [45]. Since the lifetime of SHPs is shorter on spines without synaptopodin and since synaptopodin is required for the formation of the spine apparatus, we wondered if local Ca^2+^ stores are important for SHP stability.

**Fig. 3:**
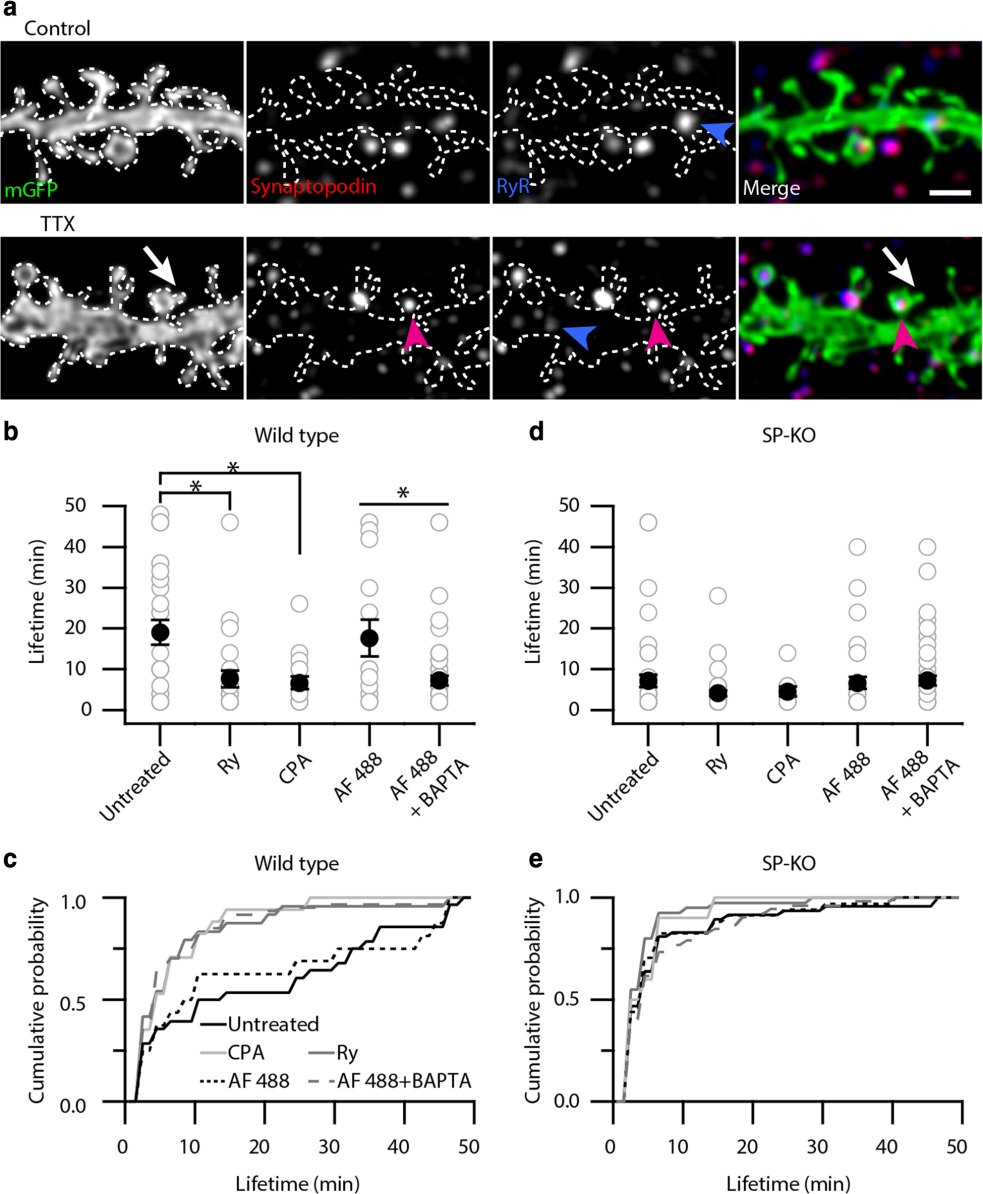
Spines forming SHPs often contain ryanodine receptors (RyRs) that colocalize with synaptopodin and disrupting Ca^2+^ release from internal stores or chelating intracellular Ca^2+^ reduces SHP lifetime. **a** Maximal intensity projections (7-8 consecutive *z* sections) of wild type slice cultures treated with control (*top*) or TTX-containing medium (*bottom*) for 2 h immunostained for synaptopodin (*red*) and RyRs (*blue*). RyRs and synaptopodin are often found colocalized, although not exclusively. Example of a spine where SHP from a TTX-treated slice (*arrow*), synaptopodin and RyR-positive punctum (*magenta arrowheads*) colocalize. Dendritically localized RyR-positive puncta can also be observed (*blue arrowheads*). Scale bar, 2 μm. **b** Mean lifetime of SHPs is reduced in wild type slices after ryanodine (Ry, 80-100 μM, with and without TTX; mean lifetime 7.67 ± 2.02 min, *n* = 24 SHPs from 10 of 10 slices; *P < 0.05, Mann-Whitney test compared to ‘untreated’) or CPA (25 μM, with and without TTX; mean lifetime, 6.71 ± 1.52 min, *n* = 17 SHPs from 9 of 12 slices; *P < 0.05, Mann-Whitney compared to ‘untreated’) treatment compared to wild type slices exposed to only control or TTX-containing medium for 1 h (19.07 ± 3.06 min, *n* = 29 SHPs from 15 of 15 slices). SHPs on wild type neurons from patched CA1 neurons with Alexa Fluor 488 (AF 488) alone formed in control and TTX solution last longer than SHPs on wild type neurons patched with BAPTA in the intracellular solution in control and TTX solution (AF 488 alone, mean lifetime, 17.63 ± 4.46 min, *n* = 16 SHPs from 8 of 8 slices; AF 488 + BAPTA, mean lifetime 7.26 ± 1.17 min, *n* = 60 SHPs from 12 of 12 slices; *P < 0.05, Mann-Whitney test). **c** Cumulative distribution of all SHP lifetimes from wild type slices. **d** Mean lifetime of SHPs is unchanged in SP-KO cultures after ryanodine (with and without TTX; 4.15 ± 0.72 min, *n* = 40 SHPs from 11 of 11 slices, *P* > 0.05, Mann-Whitney test compared to ‘untreated’) or CPA (with and without TTX; 4.60 ± 1.19 min, *n* = 10 SHPs from 5 of 10 slices, *P* > 0.05, Mann-Whitney test compared to ‘untreated’) treatment compared to SP-KO slices exposed to only control and TTX-containing medium for 1 h (SP-KO average lifetime of 7.15 ± 1.48 min). SHPs on SP-KO neurons from patched CA1 neurons with AF 488 alone formed in control and TTX solution are short-lived and not different from SHPs on SP-KO patched with both AF 488 and BAPTA in the pipette (SP-KO average lifetime of SHPs from AF 488 patched neurons of 6.71 ± 1.51 min, *n* = 34 SHPs from 6 of 6 slices; 7.27 ± 1.16 min, *n* = 52 SHPs from 10 of 10 slices; *P* > 0.05, Mann-Whitney test). In both **b** and **d** *gray circles* are individual lifetimes of SHPs and black circles are mean lifetimes ± SEMs. **e** Cumulative distribution of all SHP lifetimes from SP-KO slices

We immunostained wild type slices treated with control or TTX-containing media (2 h) for both synaptopodin and ryanodine receptors (Fig. 3a) and found that synaptopodin-positive puncta either frequently colocalized with or were adjacent to ryanodine receptor-positive puncta (Fig. 3a), as previously reported for dissociated neurons [29]. Moreover, we also found that colocalized synaptopodin and ryanodine receptor-positive puncta were found at spines with SHPs (Fig. 3a*right*). We conclude that synaptopodin is associated with ryanodine receptor-positive sources of Ca^2+^ in control and TTX-treated cultures.

To test if Ca^2+^ dynamics can stabilize SHPs, we pharmacologically altered Ca^2+^ homeostasis in hippocampal slices and combined this with time-lapse imaging of SHPs. First, we blocked Ca^2+^ release from ryanodine-sensitive stores with a high concentration of ryanodine (Ry, 80-100 μM) [39, 46]. In wild type slices, ryanodine significantly reduced the lifetime of SHPs (both from ryanodine treatment alone and ryanodine with TTX) compared to SHPs from wild type slices without ryanodine treatment (Fig. 3b). Second, we used cyclopiazonic acid (CPA, 25 μM), a sarco/endoplasmic reticulum Ca^2+^-ATPase inhibitor to deplete Ca^2+^ stores and inhibit Ca^2+^ release [47]. We found that in wild type slices, either treatment with CPA alone or CPA together with TTX reduced mean SHP lifetime compared to slices exposed to control or TTX-containing medium (Fig. 3b). Finally, to directly test whether intracellular Ca^2+^ is important for SHP stability, we patched individual CA1 pyramidal neurons and filled them with 100-150 μM Alexa Fluor 488 (AF 488, for morphological labeling) and included the Ca^2+^ chelator BAPTA (20 mM) to sequester Ca^2+^. Appropriate control experiments were performed to ensure that we would still be able to observe SHPs after patching and dye-filling neurons. We found that in wild type slices, CA1 neurons filled with AF 488 (without BAPTA) and exposed to TTX still formed SHPs (0.80 ± 0.02 SHPs per 10 μm of dendrite after 1 h of TTX; *n* = 3 slices, 62 μm of dendrite) that were stable and comparable to SHPs observed on mGFP-labeled neurons (mean lifetime, 17.63 ± 4.46 min, *n* = 16 SHPs from 8 slices from control and TTX experiments, *P* > 0.05 compared to wild type SHPs from mGFP neurons). After including BAPTA in the patch pipette, mean SHP lifetime decreased significantly to 7.26 ± 1.17 min (Fig. 3b). Moreover, the lifetime of SHPs from ryanodine, CPA or BAPTA-treated wild type slices phenocopied the lifetime of SHPs observed in SP-KO mice and their distributions were leftward-shifted compared to those from untreated wild type slices (Fig. 3c, e). Taken together, our results from wild type cultures show that internal Ca^2+^ signaling from ryanodine-sensitive stores is important for the stability of SHPs.

Next, we studied how these treatments would affect SHPs in SP-KO mice. Since these mice do not have spine apparatuses [32] – putative specialized Ca^2+^ stores [29, 34, 35] – we predicted that pharmacologically perturbing the internal Ca^2+^ concentration should have little or no effect on SHP lifetimes in these mice. Indeed, treating SP-KO hippocampal slice cultures with either ryanodine alone or ryanodine with TTX did not significantly change the mean lifetime of SHPs compared to SHPs found in SP-KO slices treated with control or TTX solution (Fig. 3d). CPA treatment alone or CPA with TTX did not significantly alter mean SHP lifetime (Fig. 3d). In addition, including BAPTA in the patch pipette had no effect on mean SHP lifetime compared to the mean lifetime of SHPs on neurons patched with only adding AF 488 to the internal solution in SP-KO slices (Fig. 3d). The distributions of SHP lifetimes after the different Ca^2+^ pharmacological treatments were similar to SHP lifetimes in SP-KO slices without treatment (Fig. 3e). Thus, inhibition of intracellular Ca^2+^ stores and the lack of synaptopodin were not additive in their effects on SHP stability. Therefore, we propose that Ca^2+^ signaling from ryanodine-sensitive stores, for example the spine apparatus, stabilizes SHPs.

### Reintroducing synaptopodin stabilizes and prolongs the lifetime of SHPs

To provide further evidence for the role of synaptopodin in the stabilization of SHPs, we studied spine dynamics in slice cultures prepared from mice expressing GFP-tagged synaptopodin on a SP-KO background. These mice were created by crossing a transgenic mouse line expressing GFP-labeled synaptopodin under the Thy1.2 promoter with the SP-KO mouse line (Thy1-GFP/SP x SP-KO mice) [42]. In these mice effects of SP-deficiency were rescued, specifically the spine apparatus was re-established in organotypic slice cultures generated from brain of these mice [39].

**Fig. 4:**
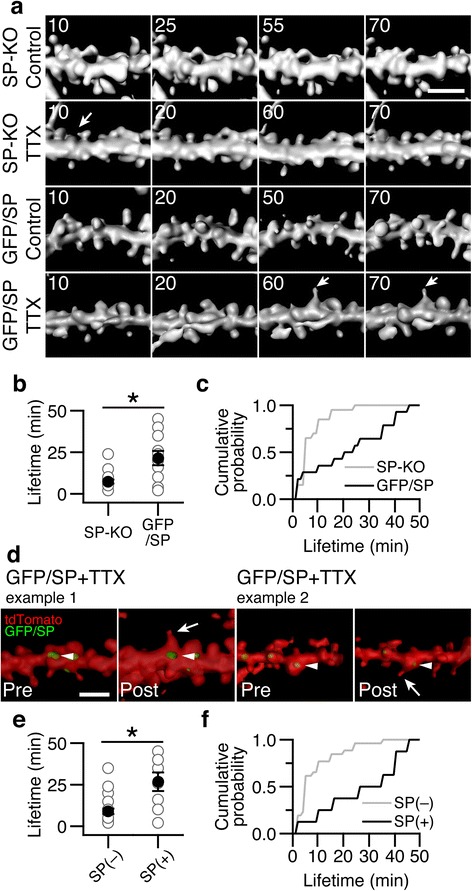
Reintroducing GFP-labeled synaptopodin into synaptopodin-deficient neurons increases SHP lifetimes. **a** Examples of small regions of dendrites from SP-KO (*top two rows*) and GFP/SP (*bottom two rows*) slices exposed to 60 min of either control or TTX-containing medium (as indicated). *Arrows* denote SHPs. Time is in minutes in top-left corner, and 10 min was the starting time point. Scale bar, 2 μm. **b** Quantification of mean lifetime of all SHPs (from both control and TTX experiments) from SP-KO and GFP/SP slices. Mean lifetime of SHPs from GFP/SP dendrites is longer (21.50 ± 4.22 min, *n* = 14 SHPs from 11 of 18 slices that formed SHPs) than SHPs formed on SP-KO dendrites (7.40 ± 1.21 min, *n* = 20 SHPs from 12 of 20 slices that formed SHPs). *P < 0.05, Mann-Whitney test. **c** Cumulative distribution of all SHP lifetimes from SP-KO (gray line) and GFP/SP (*black line*) slices. **d** Two examples of SHPs formed on spines containing GFP-labeled synaptopodin. *Left*, example 1 is the same dendrite from the GFP/SP TTX-treated slice in **a** (*last row*), just prior to the beginning (Pre) and following the end (Post) of a 60 min imaging session. *Right*, another example (example 2) of a SHP formed on a synaptopodin-positive spine. *Arrowheads* show synaptopodin (*green*), the neuron is labeled in red (tdTomato) and is rendered semi-transparent and the arrows point to the SHPs. Scale bar, 2 μm. **e** Quantification of mean lifetime of all SHPs (from both GFP/SP and SP-KO slices and control and TTX experiments) from SP-negative and SP-positive spines. Mean lifetime of SHPs on SP-positive spines is longer (26.75 ± 5.61 min, *n* = 8 SHPs from 8 of 10 slices) than SHPs formed on SP-negative spines (9.04 ± 1.64 min, *n* = 26 SHPs from 18 of 38 slices). *P < 0.01, Mann-Whitney test. In **b** and **d** *gray circles* are individual lifetimes of SHPs, and black circles are mean lifetimes ± SEMs. **f** Cumulative distribution of all SHP lifetimes from SP-negative (*gray line*) and SP-positive (*black line*) spines

To visualize CA1 pyramidal neurons, we virally transduced tdTomato into slice cultures prepared from Thy1-GFP/SP x SP-KO mice and SP-KO littermates. Many CA1 neurons were labeled with both fluorophores, allowing us to visualize both neuronal morphology (tdTomato), in particular dendrites and spines, and synaptopodin (GFP). Only few SHPs were maintained over an hour in SP-KO slices treated with either control solution or TTX (Fig. 4a). The mean lifetime of SHPs formed on SP-KO spines was 7.40 ± 1.21 min (Fig. 4b). Reintroducing synaptopodin increased the stability of new SHPs, so that after 1 h of TTX, 0.33 ± 0.09 SHPs per 10 μm of dendrite were formed (Fig. 4a; *n* = 10 slices, 247 μm of dendrite; *P* < 0.05, two-tailed, one-way ANOVA with Dunnett’s test compared to SP-KO control, SP-KO TTX and GFP/SP control. Control treated, no new SHPs in GFP/SP after 1 h; *n* = 8 slices, 197 μm of dendrite). In GFP/SP slices, the mean lifetime of SHPs was increased to 21.50 ± 4.22 min (Fig. 4b), significantly longer than the lifetime of SHPs from SP-KO slices (Fig. 4b). A clear rightward shift in the distribution of SHP lifetimes is seen when synaptopodin is reintroduced (Fig. 4c).

In order to distinguish between synaptopodin-positive and synaptopodin-negative spines in GFP/SP slices, we also imaged GFP-labeled synaptopodin prior to (Fig. 4d, *Pre*) and after (Fig. 4d, *Post*) time-lapse imaging. We found that while synaptopodin-negative spines from both GFP/SP and SP-KO slices formed protrusions with a mean lifetime of 9.04 ± 1.64 min, synaptopodin-positive spines formed protrusions lasting an average of 26.75 ± 5.61 min, significantly longer compared to synaptopodin-negative spines (Fig. 4e). Moreover, the distribution of SHP lifetimes was again shifted to the right (Fig. 4f), showing that synaptopodin-positive spines formed longer-lasting SHPs. Taken together, our results indicate that synaptopodin stabilizes dendritic SHPs.

## Discussion

The ability of the brain to rewire its microcircuit is a naturally occurring repair mechanism, which allows the CNS to ameliorate or even compensate functional deficits caused by brain lesions of any kind [2, 5, 12, 48]. However, there is still a need to understand the cellular and molecular basis of this phenomenon to optimize and time treatment strategies [49] and/or to find new targets for intervention. In the present study, we have focused on one of the mechanisms implicated in network rewiring, i.e., the formation of SHPs under conditions of network perturbance [24, 25]. We have focused on the regulatory role of the actin-modulating protein synaptopodin, which is involved in functional and structural plasticity of cortical synapses [29, 39, 50, 51]. We found that: (i) synaptopodin is associated with spines forming SHPs; (ii) spines from SP-KO hippocampus formed SHPs that were unstable; (iii) disrupting Ca^2+^ release from intracellular stores shortened protrusion lifetime and mimicked the SP-KO phenotype; and (iv) SHPs formed on spines with synaptopodin are longer lasting compared to synaptopodin-negative spines. We conclude that the presence of synaptopodin in a spine increases SHP stability, likely through Ca^2+^ release from local ryanodine-sensitive Ca^2+^ stores. The potential for rewiring within the local microcircuitry may thus be higher in spines containing synaptopodin.

### The stability of spines depends on synaptopodin, the spine apparatus and spine calcium stores

The role of synaptopodin in the CNS has been investigated during the last decade and it has been shown to play a role in different forms of plasticity at hippocampal synapses [30, 32, 39, 42, 52, 53]. At the mechanistic level, many of these biological functions are linked to the spine apparatus organelle [43, 54], which is found in some but not all spines and which is an efficient spine calcium store [29, 34, 35, 42]. Synaptopodin is an essential component of this organelle [32] and mice lacking synaptopodin exhibit single tubules of smooth ER but not its stacked and densely packed version, i.e. the “spine apparatus” [54]. Using these mice and pharmacology, we have studied the role of calcium stores in the formation and stabilization of SHPs. Both approaches revealed that the formation of SHPs does not depend on the presence of calcium stores in spines, since SHPs formed under conditions of pharmacological calcium depletion as well as in SP-KO mice. Their stability, however, was clearly affected since the lifetime of SHPs was considerably shortened under these conditions. Of note, pharmacological calcium store depletion did not have any additive effect on the lifetime of SHPs in SP-KO mice, suggesting that spine-apparatus associated ryanodine-sensitive calcium stores indeed regulate their stability.

What could be the link between the presence of a spine apparatus and SHP stability? We previously showed that SHP formation partially depends on NMDA receptor activation [24], and because NMDA receptors can be activated by single quanta of glutamate in an AMPA receptor-dependent manner [55], we propose that Ca^2+^ entering through NMDA receptors may trigger Ca^2+^-induced Ca^2+^-release important for SHP stability. Since Ca^2+^ signaling over the time frame of seconds activates Rho GTPases which, in turn, results in spine shape changes lasting tens of minutes [56, 57], the lack of Ca^2+^ signaling in synaptopodin-negative spines may fail to activate Rho GTPases [58] and, thus, SHPs would not be maintained. Although this hypothesis appears to be straightforward, other contributors to internal Ca^2+^ dynamics, namely Ca^2+^ from IP_3_-sensitive stores released through metabotropic glutamate receptor (mGluR) activation could also be involved. Indeed, while spines with ER can undergo mGluR-dependent depression, neighboring spines without ER cannot [35]. Again, Ca^2+^ release from these stores, many of which are synaptopodin-positive, were responsible for triggering depression [35]. Direct measurements of calcium in spines with and without SHPs will be needed to shed more light on the precise role of calcium stores in the context of SHP stabilization.

### Synaptopodin may regulate SHPs via actin reorganization

Synaptopodin is an actin-binding protein [26] and given that spine motility is driven by actin dynamics [22, 59], synaptopodin itself or through its interaction (s) with other actin-binding proteins may act to stabilize actin filaments within SHPs. In fact, synaptopodin binds to α-actinin [60, 61] that crosslinks and bundles actin [62]. By protecting actin filaments from disassembly, synaptopodin is also important for the sustained enlargement of dendritic spines during LTP [30, 31]. Thus, another possible pathway through which synaptopodin influences SHP stability could be via the regulation of actin remodeling.

### Synaptopodin and network rewiring

In our initial description of SHPs, we found that SHPs formed within ~10 min of iontophoretic glutamate application in a directional manner, suggesting that glutamate triggers a reaction within spines to form protrusions [24, 25]. Moreover, after triggering SHPs, we found that a subsequent glutamate application within 20 min of their formation could destabilize them. Thus, we proposed that SHPs rapidly mature over ~10 min of their initial formation [24, 25]. Our present findings support the idea of an initial phase of formation and a later phase of SHP stabilization. Spines lacking synaptopodin or spines in SP-KO animals formed SHPs that retracted within ~10 min of forming. In contrast, spines containing synaptopodin were maintained for longer time periods (>15 min). These observations, together with our pharmacological data imply that synaptopodin and/or the spine apparatus could be part of the cellular machinery required for SHP stabilization. The presence of synaptopodin/spine apparatus, by stabilizing SHPs, may lengthen the associations of SHPs with new, local presynaptic partners and allow these spines to persist for long periods. Accordingly, these spines may be more resistant to elimination during sensory deprivation [63, 64] or denervation [65, 66]. The presence of synaptopodin in a spine may thus play a critical role in brain rewiring under pathological conditions, since it will determine whether a specific spine will or will not form a new synapse during microcircuit rewiring.

### Implications for brain repair

The rewiring of neuronal networks is an important endogenous repair mechanism following neuronal damage [1, 2, 5]. Furthermore, it can be enhanced using modern rehabilitation strategies [11, 12]. As such, it warrants further investigation since strategies aimed at optimizing neuronal rewiring postlesion are undoubtedly called for. In our in vitro approach, we have modeled changes in network activity using TTX-treatment. This experimental setting allowed us to study rewiring caused by a decrease in network activity, which occurs at different levels of a network following brain damage. Using this approach, we identified synaptopodin as a molecule involved in microcircuit rewiring via SHPs.

Other aspects important for brain repair, e.g. changes at the injury site, transneuronal changes in areas of denervation [1, 2], etc., were not investigated, although they all play a role in a clinical setting. Similarly, we did not investigate posttraumatic epilepsy, a condition observed in a significant percentage of patients with brain trauma [67, 68], which may also affect rewiring. In fact, we consider the absence of such factors advantageous for unraveling cell biological mechanisms involved in brain reorganization, since too many variables, such as inflammatory signaling molecules released at an injury site (e.g., Loane and Kumar, 2016) [69], can make data interpretation very difficult. After the identification of candidate regulatory molecules, however, more complex in vivo lesioning models will be needed to demonstrate the in vivo relevance of our findings in the context of brain injury.

## Conclusions

We have studied the reorganization of brain microcircuits in vitro. Our data show that under conditions of network-perturbance (TTX-treatment) hippocampal pyramidal cells extend SHPs, which reach out toward neighboring terminals to form new synapses. Spines forming SHP exhibited more stable protrusions if the spine also contained synaptopodin. Thus, the presence of synaptopodin influences the rewiring of neuronal microcircuitries and makes the rewiring more efficient. Further studies using lesion models are now needed to show that this mechanism is relevant under in vivo conditions.

## Additional files

Additional file 1: SI Materials and Methods. (DOC 71 kb) Additional file 2: SI Figure Legend. Figure S1 CA1 neurons in slices cultures from synaptopodin-knockout (SP-KO) mice have comparable morphological and functional properties to wild type (WT) slices. a, Examples of CA1 dendrites rendered in 3D from WT and SP-KO slices. Scale bar, 2 μm. b, Quantification of spine densities, lengths and volumes from WT and SP-KO slices, expressed as percent of WT values. For spine densities, WT, *n* = 23 branches, 599 μm of dendrite from 22 slices; SP-KO, *n* = 23 branches, 645 μm of dendrite from 18 slices were analyzed. For spine lengths and volumes, WT, *n* = 1,021 spines from 22 slices; SP-KO, *n* = 1,121 spines from 18 slices were studied. c, Cumulative probability distributions of spine lengths (left) and spine volumes (right) in WT and SP-KO slice cultures. d, Example traces of mEPSCs recorded from CA1 pyramidal cells in WT (left) and SP-KO (right) slice cultures. e, Quantification of mean mEPSC amplitude (left; WT, 13.9 ± 0.38 pA and SP-KO, 13.6 ± 0.67 pA) and mean inter-mEPSC interval (right; WT, 228.37 ± 25.97 ms; SP-KO, 328.06 ± 49.14 ms). WT, *n* = 17 cells from 10 cultures; SP-KO, *n* = 15 cells from 8 cultures. f, Cumulative probability distributions of mEPSC amplitudes (left) and inter-mEPSC intervals (right) from WT and SP-KO CA1 neurons. (TIF 360 kb)
