# Supplementary material for: Rewiring neuronal microcircuits of the brain via spine head protrusions-a role for synaptopodin and intracellular calcium stores
Source: Acta Neuropathol Commun. 2016 Apr 22;4:38. doi: 10.1186/s40478-016-0311-x (PMC4840984; doi:10.1186/s40478-016-0311-x)
Supplement: Additional file 1: — SI Materials and Methods. (DOC 71 kb) [file 40478_2016_311_MOESM1_ESM.doc]

**SUPPORTING INFORMATION**

**SI Materials and Methods**

**Slice cultures.** All procedures for animal handling were carried out either according to the guidelines of the Canadian Council on Animal Care (experiments performed at McGill University, Montreal) or the German law on the use of laboratory animals (experiments performed at Goethe University, Frankfurt; approved by the animal welfare officer of Goethe-University, Faculty of Medicine). For the roller-tube method, slices of hippocampus (400 μm) were cut with a tissue chopper and placed on coverslips and adhered with a plasma clot. Coverslips with the slices were then transferred to flat bottom tubes with medium (50% (v/v) Eagle's basal medium, 25% (v/v) HBSS and 25% (v/v) horse serum) and placed in a roller-drum in a dry-air incubator (36 **°**C). Slice cultures were maintained for 3–6 weeks before use to allow for spine innervation and maturation and media were changed weekly.

For the interface cultures, entorhino-hippocampal slice cultures (300 μm) prepared with a vibratome were placed on membrane inserts (4-6 slices per insert) and then placed in six-well culture dishes with medium (50% (v/v) MEM, 25% (v/v) basal medium eagle, 25% (v/v) heat-inactivated normal horse serum, 25 mM HEPES buffer solution, 0.15% (w/v) bicarbonate, 0.65% (w/v) glucose, 0.1 mg/ml streptomycin, 100 U/ml penicillin, and 2 mM glutamax) that was adjusted to pH 7.3 and replaced every 2-3 d. The slices were grown in a humidified atmosphere with 5% CO_2_ at 35 **°**C and maintained for 18-30 d before use to allow for spine innervation and maturation .

**Mouse strains.** L15 mice express low, but consistent levels of membrane-targeted EGFP (mGFP)-positive cells within the hippocampus (under the control of Thy1.2 promoter) and were used as the wild type genotype . These slices were cultured using the roller-tube method. Synaptopodin-knockout (SP-KO) mice were crossed with L15 mice to generate mice heterozygous for the *synaptopodin* gene and expressing mGFP under the Thy1.2 promoter and then bred with other heterozygous (SP^+/–^) mice to establish homozygous synaptopodin-null mice (SP-KO; SP^–/–^) expressing mGFP. These mice were used as the SP-KO genotype and cultured with the roller-tube method.

For experiments where we visualized synaptopodin in CA1 neurons using time-lapse confocal microscopy, we used slice cultures derived from Thy1-GFP/synaptopodin mice crossed with synaptopodin-knockout mice (Thy1-GFP/SP x SP-KO) and slice cultures from their SP-KO littermates were used as controls. Slices were prepared at P3-5 and CA1 neurons were visualized by viral transduction (adeno-associated virus) of the synapsin-promoter-driven red fluorophore tdTomato at 7-10 days *in vitro*. Slices were kept in culture for an additional 10 d to allow for expression of the fluorophore before CA1 pyramidal neurons labeled for both GFP-SP and tdTomato were imaged. Age- and time-matched cultures prepared from SP-KO littermates were transfected at the same time to serve as controls. In addition, we performed time-lapse experiments on wild type slice cultures prepared on membrane inserts and transfected them with the tdTomato virus to determine whether tdTomato sufficiently labels dendrites and spines to visualize SHPs. We found 0.10 ± 0.10 SHPs per 10 μm of dendrite (*n* = 3 slices, 81 μm of dendrite) after 1 h in control medium. Similar to our previous findings , 1 h of 1 μM TTX increased the appearance of SHPs on tdTomato neurons (0.52 ± 0.16 SHPs per 10 μm of dendrite; *n* = 3 slices, 80 μm of dendrite).

**Immunostaining and static imaging.** Briefly, cultures were fixed for 1-3 h in 4% (w/v) paraformaldehyde in 0.1 M phosphate buffer (PB; pH 7.4) at room temperature. Slices were then pre-incubated with a blocking/permeabilizing solution (0.1 M PB supplemented with 1.5% (v/v) heat-inactivated horse serum and 0.4% (v/v) Triton X-100) overnight at 4 **°**C. Next, primary antibodies were applied in 0.1 M PB containing 1.5% (v/v) horse serum and 0.4% (v/v) Triton X-100 for 5 d at 4 **°**C. Primary antibodies used include rabbit anti-synaptopodin (1:500; SE-19, Sigma-Aldrich, St. Louis, MO; antibody specificity previously established ) and mouse anti-ryanodine receptor (1:200; C3-33, Calbiochem, EMD Millipore, Billerica, MA). After repeated washes with 0.1 M PB supplemented with 1.5% (v/v) horse serum (including an overnight wash at 4 **°**C), slices were incubated with species-matched secondary antibodies (anti-rabbit Alexa Fluor 594; 1:250, anti-mouse Alexa Fluor 633; 1:250; Molecular Probes, Eugene, OR; or anti-mouse DyLight 649, 1:400; Jackson ImmunoResearch, West Grove, PA) for 3-4 h at room temperature. Slices were washed in 0.1 M PB overnight at 4 **°**C and then mounted with Dako Fluorescent Mounting Media (Dako Canada, Burlington, ON) onto microscope slides.

Images of immunostained cultures were taken with a Leica TCS SP2 scanhead (Leica Microsystems, Wetzlar, Germany) connected to an upright microscope (DM 6000B) using either a 40× oil objective (HCX PL APO 1.25 NA, Leica) or a 63× oil objective (HCX PL APO 1.40 NA, Leica). EGFP was imaged using a 488-nm Ar laser line, red fluorophores with a 543-nm HeNe laser line, and far-red fluorophores with a 633-nm HeNe laser line and acquired sequentially. Line and/or frame averaging (2-8 times) were used to improve the signal-to-noise ratio. Dendritic segments of second- or third-order (both apical and basal branches) were imaged with voxel dimensions of ~46 x 46 x 300-400 nm. Images were processed as described below (see **3D Image Reconstruction and Analysis**).

**Time-lapse confocal imaging.** Slice cultures prepared according to the roller-tube method were placed into a heated (30-32 ºC) recording chamber of an upright microscope (DM LFSA, Leica or Axio Examiner.Z1, Zeiss) and perfused with Tyrode solution (consisting of (in mM): 137 NaCl, 2.7 KCl, 2.5 CaCl_2_, 2 MgCl_2_, 11.6 NaHCO_3_, 0.4 NaH_2_PO_4_, and 5.6 D-glucose (pH 7.4) and gassed with 95% O_2_/5% CO_2_.). The confocal scanhead was a Leica TCS SP2 or a Zeiss LSM 710. EGFP was imaged using a 488-nm Ar laser line.

For experiments using slice cultures prepared according to the interface method (rescue experiments), slices were transferred to a heated (30-32 ºC) recording chamber of an upright microscope (Axioskop 2 FS, Zeiss) and perfused with Tyrode solution (as above). The confocal scanhead was a Zeiss LSM 5 PASCAL. tdTomato was imaged using a 543-nm HeNe laser line and GFP was imaged with a 488-nm Ar laser line and were acquired sequentially when performing two-color imaging.

In a series of experiments, instead of imaging transgenically GFP-labeled neurons, we patched individual CA1 neurons with pipettes containing an internal solution (see *Electrophysiology*) with 100-150 μM Alexa Fluor 488 to label cell morphology and imaged with a 488-nm Ar laser line. We waited 10-15 min for the neuron to fill, and then began time-lapse imaging of dendritic branches (see below). To test if internal Ca^2+^ is important for SHP, we added the Ca^2+^ chelator BAPTA tetrapotassium salt (20 mM, Sigma-Aldrich) to the pipette in addition to Alexa Fluor 488 and waited 10-15 min for both the chelator and dye to fill the neuron. We performed control experiments without BAPTA to determine how dye-filling can affect SHPs compared to mGFP (see **Ca^2+^ release from ryanodine-sensitive stores increases SHP lifetime**).

Dendritic segments from tertiary and secondary apical or basal branches were imaged with voxel dimensions of ~46 x 46 x 300-350 nm (512 x 512) or 92 x 92 x 300-350 nm (256 x 256) and line averaged twice using a 63× water immersion long working distance lens (HCX APO L U-V-I, 0.9 NA, Leica or W Plan-APOCHROMAT, 1.0 NA, Zeiss with the LSM 710 or Achroplan, 0.95 NA, Zeiss with the LSM PASCAL 5). Additional optical sections were taken above and below the structure of interest to allow for any changes in the structure with time. Once the images were captured, the area of interest was cropped and further processed for 3D reconstruction and analysis.

**3D Image Reconstruction and Analysis.** Image stacks were deconvolved using Huygens Essential software (Scientific Volume Imaging, Hilversum, The Netherlands) with a full maximum likelihood extrapolation algorithm. Volume rendering and quantification were carried out using Imaris x64 software (Bitplane AG, Zurich, Switzerland). No filtering or resampling was performed. Each channel was thresholded independently, and image analyses were performed under visual control to determine thresholds that subtracts background noise and takes into account neuronal structure. All analyses were performed on the entire 3D or 4D data set. 3D isosurfaces were rendered using the Surpass tool in Imaris, and the same parameters were used for all time points of an experimental series.

SHPs were quantified by finding pointy structures emerging from spine heads that were ≥0.5 μm in length. During image processing, the images were compared with the original raw data to make sure that no structures were introduced that were not seen in the original data series or that structures present in the original data series were not removed. To calculate SHP lifetime, every SHP that appeared on a spine (both *de novo* and present at the start of imaging) was counted and if SHPs appeared in only one time frame, then it was assumed that these SHPs had lifetimes corresponding to the interval between *z* stacks (2 or 5 min). Because stack acquisition took ~30 s (usually 24 stacks for 60 min imaging experiment, with a time interval of 2 min), then the maximum lifetime is 48 min (48 min of imaging, not counting the 12 min of image acquisition). For live imaging experiments, we quantified one dendrite per cell, per slice.

Because creating maximum intensity projections from entire *z* stacks could potentially make synaptopodin- and/or ryanodine receptor-positive puncta above or below our dendrites or spines of interest appear to be within those structures, to visualize synaptopodin- and/or ryanodine receptor-positive puncta only within our structures of interest, we masked (using the green, mGFP-dendrite channel) and retained only red, synaptopodin-positive puncta and/or blue, ryanodine receptor-positive puncta that colocalized with the green channel. We then located SHPs on spines that contained or lacked synaptopodin puncta and/or ryanodine receptor. We regarded spines as synaptopodin-positive if they clearly contained punctum in the spine head or neck, as well as if a punctum was within a 0.5 μm radius of the spine (like at the base/dendrite).

We identified and quantified spines using a semi-automated method in Imaris Surpass (Bitplane). We manually traced individual spines using the Filament Tracer function to calculate spine density and the spine detection/classification program automatically detected apparent spine lengths and volumes.

**Electrophysiology.** Slice cultures were transferred to a heated (30-32 ºC) recording chamber of an upright microscope (DM LFSA, Leica, HCX APO L U-V-I 40× 0.8 NA water immersion objective, or BX51WI, Olympus, XLUMPlanF1 20× 0.95 NA water immersion objective) and continually perfused with Tyrode solution containing (in μM): 1 TTX, 25 CPP, 40 bicuculline or 50 picrotoxin, and 5 CGP 55845 in order to isolate AMPA receptor-mediated miniature excitatory postsynaptic currents (mEPSCs). Whole-cell voltage clamp recordings were obtained from CA1 pyramidal neurons in either wild type or SP-KO slices held at –60 mV with an Axopatch 200A amplifier (Molecular Devices). Borosilicate patch pipettes (3-6 MΩ) were filled with (in mM) 120 K-gluconate, 1 EGTA, 10 HEPES, 5 MgATP, 0.5 Na_2_GTP, 5 NaCl, 5 KCl and 10 phosphocreatine K_2_ (pH 7.2-7.3 with KOH and 280-295 mOsm). When the Ca^2+^ chelator BAPTA tetrapotassium salt (20 mM) was included in the intracellular solution for imaging experiments, the concentration of K-gluconate was reduced to ~100 mM. Access resistance was monitored with brief test pulses at regular intervals (2-3 min) throughout the experiment. Access resistance was 10-13 MΩ and data were discarded if the resistance deviated more than 20% through the course of the experiment. After the holding current had stabilized, data were recorded at a sampling frequency of 10 kHz and filtered at 2 kHz for 10 to 15 min.

All mEPSCs were detected offline using Mini Analysis Software (Synaptosoft, Decatur, GA, USA). The amplitude threshold for mEPSC detection was set at four times the root-mean-square value of a visually event-free recording period and 300-600 events per cell were analyzed and used to determine mean values and cumulative probability plots.

**Reagents.** Alexa dyes and antibodies were obtained from Molecular Probes (Eugene, OR). All other reagents were purchased from Tocris Bioscience (Ellisville, MO), except TTX, which was purchased from Alomone Labs (Jerusalem, Israel).

**SI Figure Legend**

**Fig. S1. CA1 neurons in slices cultures from synaptopodin-knockout (SP-KO) mice have comparable morphological and functional properties to wild type (WT) slices.**

**a**, Examples of CA1 dendrites rendered in 3D from WT and SP-KO slices. Scale bar, 2 μm. **b**, Quantification of spine densities, lengths and volumes from WT and SP-KO slices, expressed as percent of WT values. For spine densities, WT, *n* = 23 branches, 599 μm of dendrite from 22 slices; SP-KO, *n* = 23 branches, 645 μm of dendrite from 18 slices were analyzed. For spine lengths and volumes, WT, *n* = 1,021 spines from 22 slices; SP-KO, *n* = 1,121 spines from 18 slices were studied. **c**, Cumulative probability distributions of spine lengths (left) and spine volumes (right) in WT and SP-KO slice cultures. **d**, Example traces of mEPSCs recorded from CA1 pyramidal cells in WT (left) and SP-KO (right) slice cultures. **e**, Quantification of mean mEPSC amplitude (left; WT, 13.9 ± 0.38 pA and SP-KO, 13.6 ± 0.67 pA) and mean inter-mEPSC interval (right; WT, 228.37 ± 25.97 ms; SP-KO, 328.06 ± 49.14 ms). WT, *n* = 17 cells from 10 cultures; SP-KO, *n* = 15 cells from 8 cultures. **f**, Cumulative probability distributions of mEPSC amplitudes (left) and inter-mEPSC intervals (right) from WT and SP-KO CA1 neurons.

**SI References:**

1. Verbich D, Prenosil GA, Chang PKY, Murai KK, & McKinney RA (2012) Glial glutamate transport modulates dendritic spine head protrusions in the hippocampus. *Glia* 60(7):1067-1077.

2. Mateos JM*, et al.* (2007) Synaptic modifications at the CA3-CA1 synapse after chronic AMPA receptor blockade in rat hippocampal slices. *J. Physiol.* 581(1):129-138.

3. Vlachos A*, et al.* (2012) Repetitive magnetic stimulation induces functional and structural plasticity of excitatory postsynapses in mouse organotypic hippocampal slice cultures. *J. Neurosci.* 32(48):17514-17523.

4. Del Turco D & Deller T (2007) Organotypic entorhino-hippocampal slice cultures—a tool to study the molecular and cellular regulation of axonal regeneration and collateral sprouting in vitro. *Methods Mol. Biol.* 399:55-66.

5. De Paola V, Arber S, & Caroni P (2003) AMPA receptors regulate dynamic equilibrium of presynaptic terminals in mature hippocampal networks. *Nat. Neurosci.* 6(5):491-500.

6. Deller T*, et al.* (2003) Synaptopodin-deficient mice lack a spine apparatus and show deficits in synaptic plasticity. *Proc. Natl. Acad. Sci. USA* 100(18):10494-10499.

7. Vlachos A*, et al.* (2013) Synaptopodin regulates denervation-induced homeostatic synaptic plasticity. *Proc. Natl. Acad. Sci. USA* 110(20):8242-8247.

8. Stoppini L, Buchs PA, & Muller D (1991) A simple method for organotypic cultures of nervous tissue. *J. Neurosci. Methods* 37(2):173-182.

9. Richards DA*, et al.* (2005) Glutamate induces the rapid formation of spine head protrusions in hippocampal slice cultures. *Proc. Natl. Acad. Sci. USA* 102:6166-6171.

10. Panatier A*, et al.* (2011) Astrocytes are endogenous regulators of basal transmission at central synapses. *Cell* 146(5):785-798.
