# Supplementary figures and images for: Rewiring neuronal microcircuits of the brain via spine head protrusions-a role for synaptopodin and intracellular calcium stores
Source: Acta Neuropathol Commun. 2016 Apr 22;4:38. doi: 10.1186/s40478-016-0311-x (PMC4840984; doi:10.1186/s40478-016-0311-x)

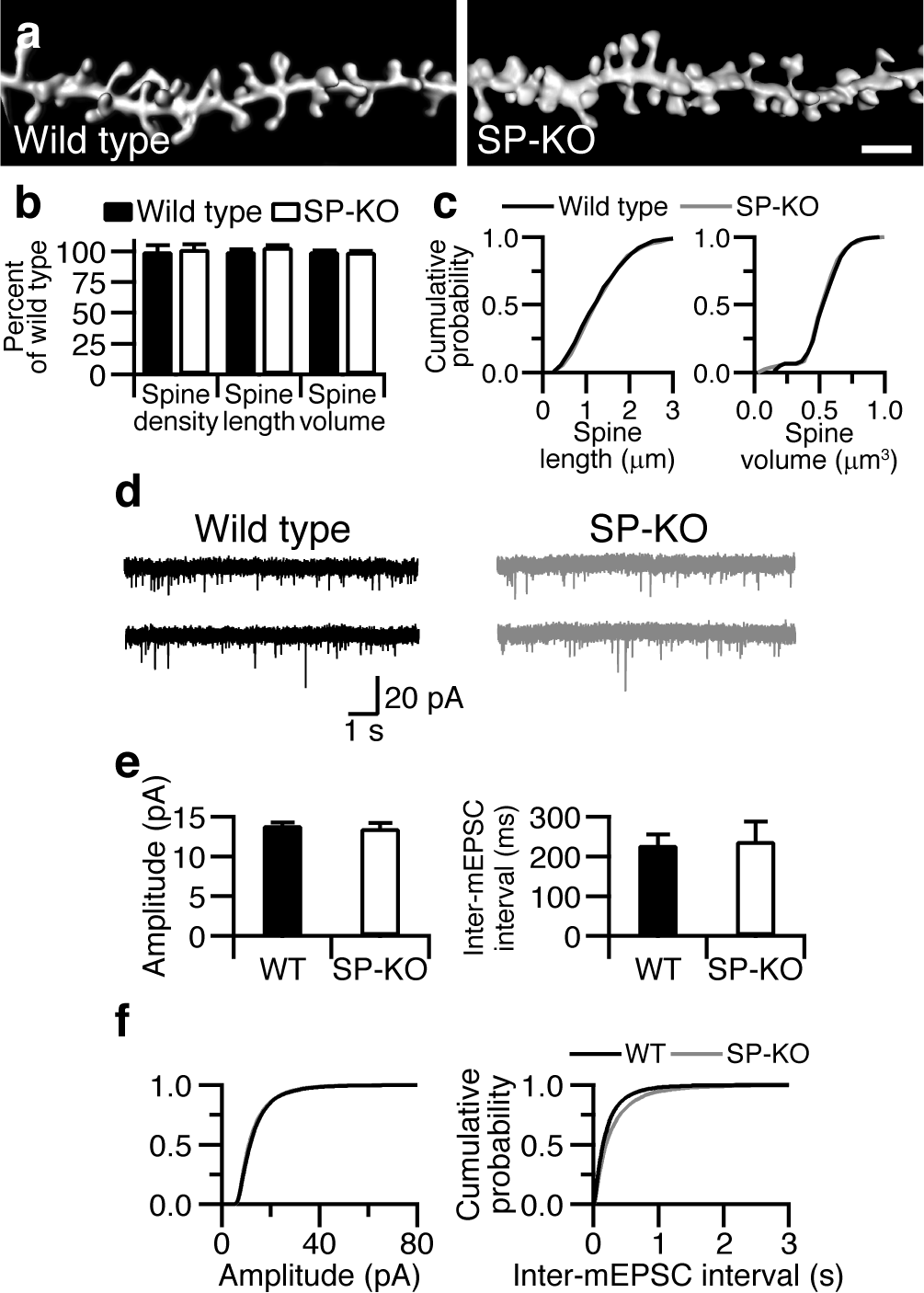

Supplement: Additional file 2: — SI Figure Legend. Figure S1 CA1 neurons in slices cultures from synaptopodin-knockout (SP-KO) mice have comparable morphological and functional properties to wild type (WT) slices. a, Examples of CA1 dendrites rendered in 3D from WT and SP-KO slices. Scale bar, 2 μm. b, Quantification of spine densities, lengths and volumes from WT and SP-KO slices, expressed as percent of WT values. For spine densities, WT, n = 23 branches, 599 μm of dendrite from 22 slices; SP-KO, n = 23 branches, 645 μm of dendrite from 18 slices were analyzed. For spine lengths and volumes, WT, n = 1,021 spines from 22 slices; SP-KO, n = 1,121 spines from 18 slices were studied. c, Cumulative probability distributions of spine lengths (left) and spine volumes (right) in WT and SP-KO slice cultures. d, Example traces of mEPSCs recorded from CA1 pyramidal cells in WT (left) and SP-KO (right) slice cultures. e, Quantification of mean mEPSC amplitude (left; WT, 13.9 ± 0.38 pA and SP-KO, 13.6 ± 0.67 pA) and mean inter-mEPSC interval (right; WT, 228.37 ± 25.97 ms; SP-KO, 328.06 ± 49.14 ms). WT, n = 17 cells from 10 cultures; SP-KO, n = 15 cells from 8 cultures. f, Cumulative probability distributions of mEPSC amplitudes (left) and inter-mEPSC intervals (right) from WT and SP-KO CA1 neurons. (TIF 360 kb) [file 40478_2016_311_MOESM2_ESM.tif]
